# Mechanical power ratio threshold for ventilator-induced lung injury

**DOI:** 10.1186/s40635-024-00649-0

**Published:** 2024-07-30

**Authors:** Rosanna D’Albo, Tommaso Pozzi, Rosmery V. Nicolardi, Mauro Galizia, Giulia Catozzi, Valentina Ghidoni, Beatrice Donati, Federica Romitti, Peter Herrmann, Mattia Busana, Simone Gattarello, Francesca Collino, Aurelio Sonzogni, Luigi Camporota, John J. Marini, Onnen Moerer, Konrad Meissner, Luciano Gattinoni

**Affiliations:** 1 Department of Anesthesiology, University Medical Center Göttingen, Göttingen, Germany; 2 Department of Medical and Surgical Sciences, Alma Mater Studiorum, University of Bologna, Bologna, Italy; 3 Department of Health Sciences, University of Milan, Milan, Italy; 4 IRCCS San Raffaele Scientific Institute, Milan, Italy; 5 Department of Health Sciences, Section of Anesthesiology, Intensive Care and Pain Medicine, University of Florence, Florence, Italy; 6 Department of Anesthesia, Intensive Care and Emergency, “City of Health and Science” Hospital, Turin, Italy; 7 Department of Pathology, ASST Bergamo Est, Seriate, Italy; 8 Department of Adult Critical Care, Guy’s and St. Thomas’ NHS Foundation Trust, Health Centre for Human and Applied Physiological Sciences, London, UK; 9 Department of Pulmonary and Critical Care Medicine, University of Minnesota and Regions Hospital, St. Paul, Minnesota USA

## Abstract

**Rationale:**

Mechanical power (MP) is a summary variable incorporating all causes of ventilator-induced-lung-injury (VILI). We expressed MP as the ratio between observed and normal expected values (MP_ratio_).

**Objective:**

To define a threshold value of MP_ratio_ leading to the development of VILI.

**Methods:**

In a population of 82 healthy pigs, a threshold of MP_ratio_ for VILI, as assessed by histological variables and confirmed by using unsupervised cluster analysis was 4.5. The population was divided into two groups with MP_ratio_ above or below the threshold.

**Measurements and main results:**

We measured physiological variables every six hours. At the end of the experiment, we measured lung weight and wet-to-dry ratio to quantify edema. Histological samples were analyzed for alveolar ruptures, inflammation, alveolar edema, atelectasis. An MP_ratio_ threshold of 4.5 was associated with worse injury, lung weight, wet-to-dry ratio and fluid balance (all *p* < 0.001). After 48 h, in the two MP_ratio_ clusters (above or below 4.5), respiratory system elastance, mean pulmonary artery pressure and physiological dead space differed by 32%, 36% and 22%, respectively (all *p* < 0.001), being worse in the high MP_ratio_ group. Also, the changes in driving pressure, lung elastance, pulmonary artery occlusion pressure, central venous pressure differed by 17%, 64%, 8%, 25%, respectively (all *p* < 0.001).

**Limitations:**

The main limitation of this study is its retrospective design. In addition, the computation for the expected MP in pigs is based on arbitrary criteria. Different values of expected MP may change the absolute value of MP ratio but will not change the concept of the existence of an injury threshold.

**Conclusions:**

The concept of MP_ratio_ is a physiological and intuitive way to quantify the risk of ventilator-induced lung injury. Our results suggest that a mechanical power ratio > 4.5 MP_ratio_ in healthy lungs subjected to 48 h of mechanical ventilation appears to be a threshold for the development of ventilator-induced lung injury, as indicated by the convergence of histological, physiological, and anatomical alterations. In humans and in lungs that are already injured, this threshold is likely to be different.

**Supplementary Information:**

The online version contains supplementary material available at 10.1186/s40635-024-00649-0.

## Introduction

Over recent decades, research in mechanical ventilation has highlighted the potential harms associated with its use and described the mechanisms of injury through excessive pressure (*barotrauma* [1]), volume (*volutrauma* [2]), opening and closing of lung units (*atelectrauma* [3]), and subsequent inflammatory responses (*biotrauma*). Although these mechanisms collectively contribute to ventilator-induced lung injury (VILI), current ventilation strategies primarily focus on optimizing individual components of ventilation such as limiting plateau pressure [4], driving pressure [5], tidal volume and strain [6], or setting sufficient PEEP to obtain an “open-lung” approach [7]. In clinical practice, several co-factors play a role in the mortality associated with a specific ventilation strategy.

To fully understand the mechanism of VILI and quantify its effects, an experimental approach is required, in which the transition from healthy to injured lungs depends only on the applied mechanical ventilation. In recent years, we proposed the mechanical power (MP, *i.e.,* the inflation energy delivered to the respiratory system per unit of time) as a comprehensive variable indicative of VILI development [8]. The concept of *ergotrauma* [9] was explored by varying tidal volume, PEEP, or respiratory rate across several animal studies [10–12].

However, a challenge with MP is its normalization, i.e., expressing MP per unit size of a biological variable. The same mechanical power, when applied to different animals, may be safe or harmful, depending on individual factors such as animal size, respiratory mechanics, or metabolic load. In analogy with the ventilatory ratio [13], we formulated the mechanical power ratio (MP_ratio_) as the quotient of actual MP delivered, to that expected in normal pigs of comparable body weight under anesthesia.

An unresolved issue in VILI is its practical definition and quantification. The pathological and physiological manifestations of VILI (e.g., edema, alveolar/septal ruptures, microthrombosis, fibrosis, etc*.*) mirror the natural progression of unresolved acute respiratory distress syndrome (ARDS). Consequently, in clinical context, the presence of VILI is inferred from the difference in measurable outcomes (e.g., mortality) when different ventilatory strategies are employed (e.g., ARMA trial [5]). However, the exact mechanisms leading to increased mortality cannot be described or quantified.

In this study, we retrospectively calculated the MP_ratio_ in animals subjected to three distinct experiments of harmful mechanical ventilation designed to produce VILI by applying for 48 h an MP that ranged from 2.6 to 63.4 J min^−1^ (0.1–2.3 J min^−1^ kg^−1^) [10–12]. The aim was to explore, in previously healthy pigs, whether a specific MP_ratio_ threshold exists for VILI induction during 48 h of mechanical ventilation, and which physiological variables are associated with the anatomical and histological changes attributed to the development of VILI.

## Material and methods

See Online Supplement for further details.

### Study population

We included 82 female domestic pigs enrolled in previous experimental studies [10–12]. The pooled data from each experiment are summarized in Table 1. In summary the experiment 1 showed that the VILI was the same for a given mechanical power regardless of the variations of each individual component. In group 1 the mechanical power was reached primarily by increasing tidal volume, in group 2 by increasing respiratory rate and in group 3 by increasing PEEP. Experiment 2 was designed to identify a “safe value of mechanical power” below which no lung lesions could be observed. Experiment 3 was designed to determine if the retardation of expiratory flow in order to reduce the energy dissipation in the lung parenchyma during the expiratory phase was associated with a reduction in VILI. As shown in Table 1, the different experiments covered a wide range of ventilatory settings and mechanical power.

**Table 1:** Ventilatory settings and mechanical power of the study population

	Experiment 1 [10]			Experiment 2 [11]			Experiment 3 [12]	
	High Vt Group 1	High RR Group 2	High PEEP Group 2	MP 2 J/min Group 1	MP 7 J/min Group 2	MP 12 J/min Group 3	Uncontrolled expiration Group 1	Controlled expiration Group 2
Pigs (*n*)	14	14	14	6	6	6	11	11
Vt (ml/kg)	32.7 ± 3.8	12.5 ± 2.4	13.4 ± 1.4	6.9 ± 0.3	9.7 ± 0.7	11.5 ± 1.1	19.2 ± 1.9	19.4 ± 1.0
RR (breath per minute)	11 ± 5	40 ± 1	16 ± 6	21.3 ± 0.8	20.8 ± 1.5	21.0 ± 1.5	9.2 ± 1.8	8.4 ± 0.5
PEEP (cmH_2_O)	5.3 ± 0.9	8.3 ± 1.5	24 ± 1.1	4.2 ± 0.2	4.2 ± 0.2	4.1 ± 0.2	5.6 ± 0.2	5.3 ± 0.3
MP (J/min)	20.8 ± 7.9	22.2 ± 8.5	21.3 ± 8.5	2.9 ± 0.2	7.4 ± 0.7	11.7 ± 0.8	8.5 ± 0.9	8.4 ± 0.6

*Vt* tidal volume, *RR* respiratory rate, *PEEP* positive end-expiratory pressure, *MP* mechanical power applied in the three experiments from which the population of the present study has been derived

### Mechanical power ratio

Mechanical power ratio was computed as the ratio of the actual mechanical power to the expected one. The mechanical power (actual and expected) was computed using the following formula [8]:

$$MP =0.098\times RR\times \left\{{{V}_{T}}^{2}\times \left[\frac{1}{2}\times {EL}_{rs}+RR\times \frac{1+I:E}{60\times I:E}\times {R}_{aw}\right]+{V}_{T}\times PEEP\right\},$$

where MP is the mechanical power, RR is the respiratory rate, *V*_T_ is the tidal volume, EL_rs_ is the respiratory system elastance, *I*:*E* is the inspiratory/expiratory ratio, *R*_aw_ is the airways resistance and PEEP is the positive end-expiratory pressure. The actual mechanical power was computed using the actual data measured during the experiment. The expected mechanical power was computed using the physiological normal values according to De Robertis et al. [14]: respiratory rate (20 breath min^−1^), tidal volume (10 mL kg^−1^), respiratory system elastance (0.75 cmH_2_O L^−1^ kg^−1^), I:E (1:2), airways resistance (3.9 cmH_2_O L^−1^ kg^−1^), PEEP (0 cmH_2_O).

The mechanical power ratio was than computed as the ratio of actual to expected MP:

$${\text{MP}}_{\text{ratio}} =\frac{{\text{MP}}_{\text{actual}}}{{\text{MP}}_{\text{expected}}}.$$

### Experimental protocol

The animal preparation, instrumentation, the number of variables and the timing of collection were the same in all three experiments. Under anesthesia, all animals, were instrumented with esophageal balloon, central venous, pulmonary, and femoral arterial and urinary catheters. The following variables were measured at baseline, 0.5 h after protocol initiation and thereafter every 6 h until the 48th hour.

#### Gas exchange

PO_2_, PCO_2_ and pH were measured both in the arterial and in mixed venous blood sampled from the pulmonary artery catheter. Venous admixture was calculated using the standard formula.

#### Respiratory system mechanics

We measured all standard indicators of respiratory system mechanics, including its chest wall and lung components. Aerated lung volumes (by helium gas dilution), elastances and lung stress were measured according to standard formulae.

#### Hemodynamics

We measured arterial, mixed venous, and wedge pressures (mmHg). Cardiac output was determined by transpulmonary thermodilution system and calculated by the reverse Fick’s method.

### Gross pathology and histology assessment

The wet-to-dry ratio was measured in tissue samples taken immediately after death from liver, kidney, muscle, bowel (1 sample for each organ) and from the basal, middle and apical regions of both lungs (1 sample for each lung region). All samples were immediately weighed (wet) then stored to 50 °C for 24 h, then weighed again (dry). Wet-to-dry ratio was computed as the ratio between the wet tissue to the dry tissue weights.

The lungs were fixed in formalin; after 1 week, 20 samples (10 for each lung) were collected. Samples were first fixed in formalin 10% for 48 h and then paraffin embedded; histological sections of 3 microns thickness were stained by hematoxylin and eosin. Each slide was examined using a microscope at different magnifications.

**Fig. 1:**
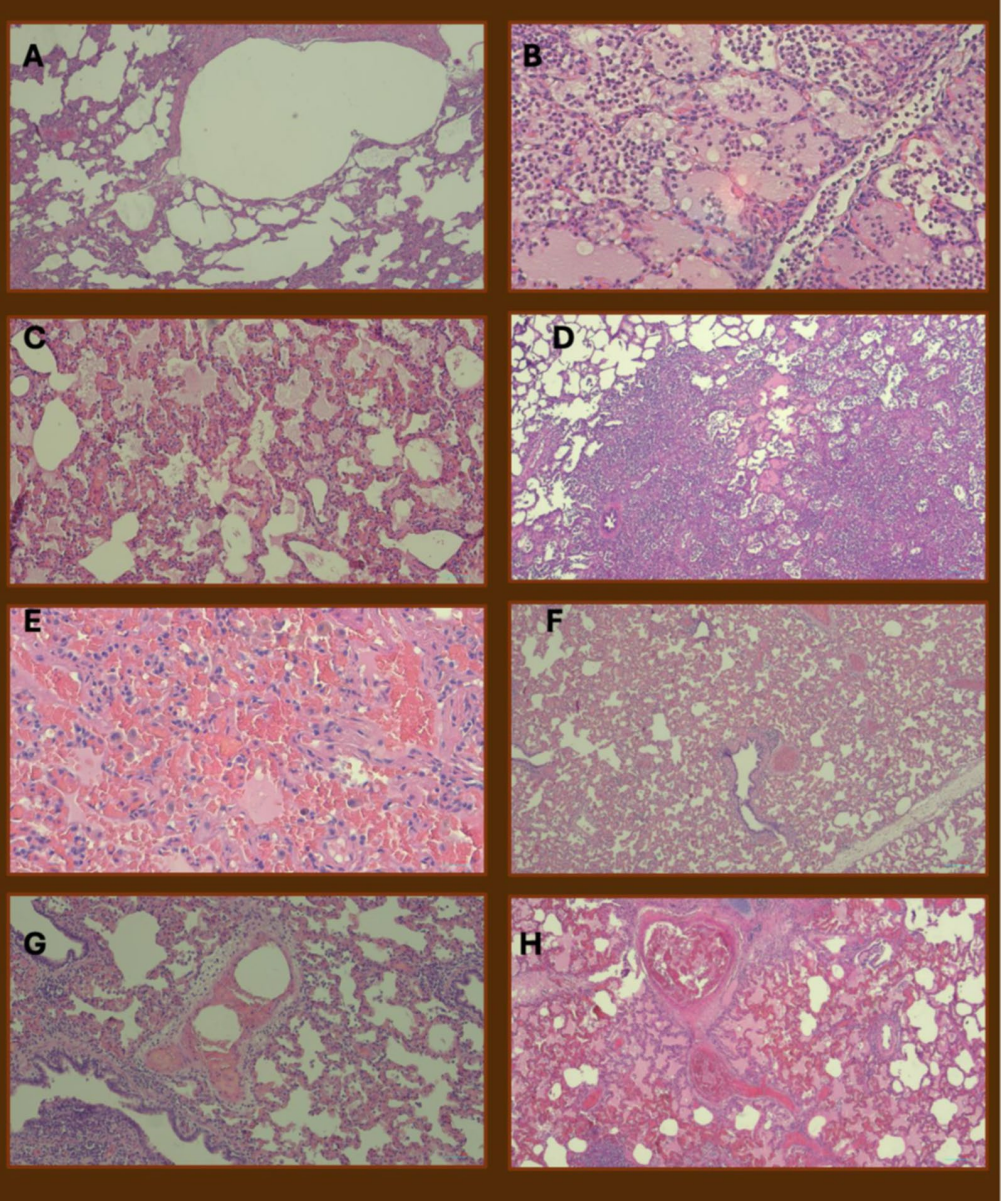
Histological findings. **A** Severely damaged lung parenchyma with massive alveolar ruptures (H&E, 40×). **B** Diffuse interalveolar exudation by neutrophils (H&E, 100×). **C** Lung parenchyma characterized by severe septal blood congestion associated with interalveolar edema (H&E, 40×). **D** Extensive lung atelectasis involving almost the whole parenchyma in histological section; note also emphysematous area in the left upper corner of the microphotograph (H&E, 40×). **E** Massive parenchymal blood septal congestion with interalveolar hemorrhages (H&E, 100×). **F** Extensive blood congestion of septa and vessel of different size (H&E, 40×). **G** Medium sized artery with edema and focal inflammatory infiltration of the wall (H&E, 40×). **H** Medium size artery with occlusive recent thrombosis of the lumen; note also marked parenchymal congestion and interalveolar edema (H&E, 40×)

Two independent pathologists, blinded to the experimental group assignment, classified each sample for the following alterations: alveolar ruptures, inflammation, alveolar edema, atelectasis, intra-alveolar hemorrhage, vascular congestion, perivascular edema, intravascular thrombi (see Fig. 1). The presence of given alterations was expressed according to the prevalence at which the alteration was observed in the field and score assigned as follow: for prevalence of 0–0.25 was assigned a score of 2, 0.25–0.5 a score of 4, 0.5–0.75 a score of 8 and 0.75–1 a score of 16. The non-linearity of the score was arbitrary decided before initiation of the study to underline the progressive increase of the lesions’ severity.

### Statistical analysis

Continuous variables are reported as median [IQR], categorical variables as % (number). To assess the pattern of distribution of histological variables, the population was arbitrarily divided into deciles of MP_ratio_ each containing an equal number of animals. Possible clusters of histological variables related to VILI were identified by an unsupervised machine learning model, based on k-means clustering. The optimal number of clusters was selected according to the Silhouette method. To identify an MP_ratio_ threshold for VILI development, a logistic regression was performed with MP_ratio_ as predictor and clusters as outcome variables. Two-way analysis of variance (ANOVA) was applied to investigate the time-course of variables, with MP_ratio_ as *between* effect and time as *within* effect; *post hoc* tests were performed by pairwise Student’s T tests. A *p* value < 0.05 was considered as statistically significant. The analysis was performed using RStudio (RStudio Team (2020). RStudio: Integrated Development for R. RStudio, PBC, Boston, MA, URL http://www.rstudio.com/).

### Mechanical power threshold finding process

**Fig. 2:**
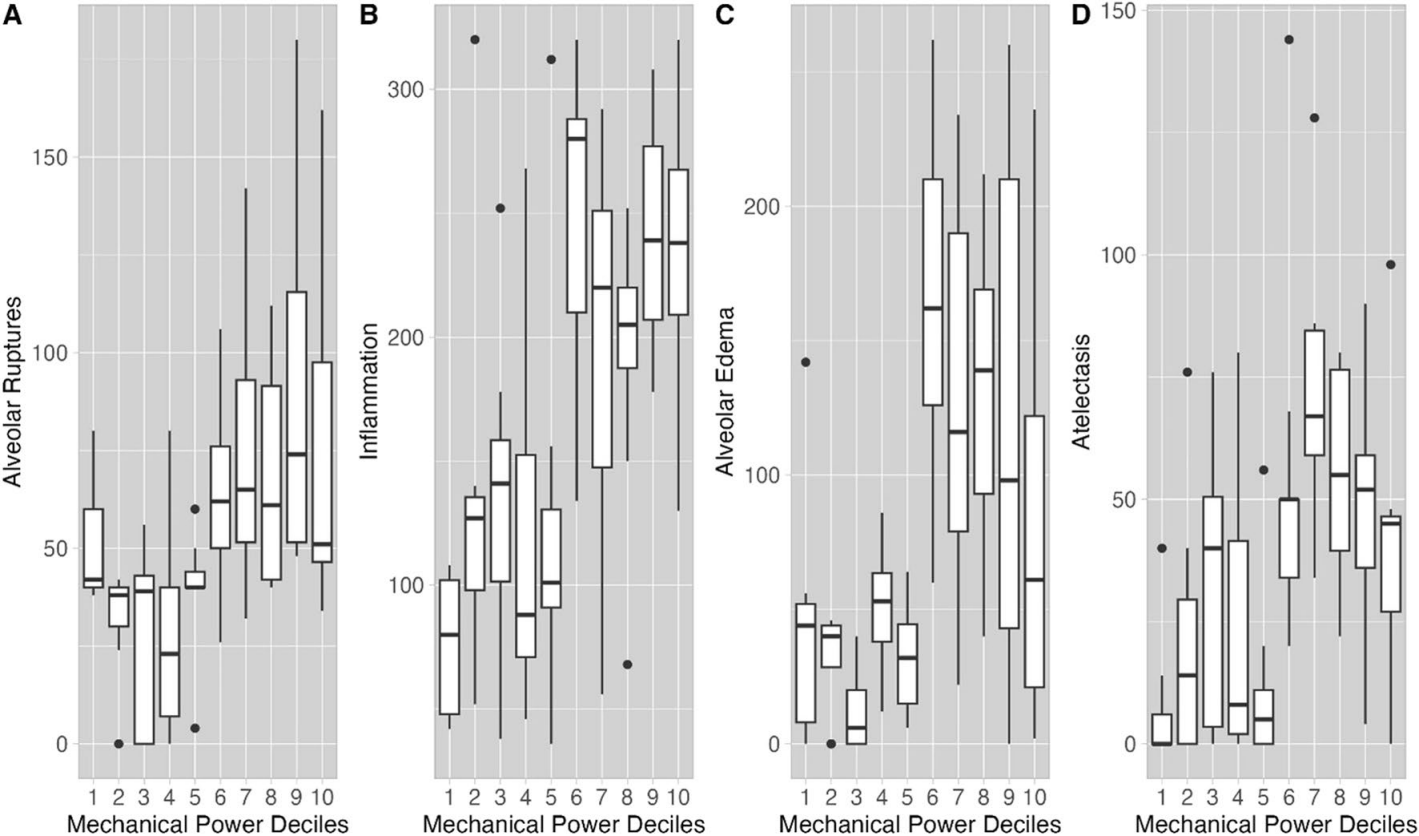
Histological damage and mechanical power ratio. Histological score for alveolar ruptures (**A**), inflammation (**B**), alveolar edema (**C**) and atelectasis (**D**) as a function of mechanical power ratio. This has been expressed in deciles (*x*-axis) including 8–9 animals each, whose upper limits are the following: decile 1: MP_ratio_ 2.40; decile 2: MP_ratio_ 2.94; decile 3: MP_ratio_ 3.12; decile 4: MP_ratio_ 3.54; decile 5: MP_ratio_ 4.46; decile 6: MP_ratio_ 6.13; decile 7: MP_ratio_ 7.28; decile 8: MP_ratio_ 10.3; decile 9: MP_ratio_ 12.5; decile 10: MP_ratio_ 24.6. As shown, upon visual inspection, at MP_ratio_ greater than 4.46 (rounded to 4.5, upper limit of the fifth decile), the histological scores markedly increased, suggesting a more severe parenchymal damage (see also Supplement, Table E1)

To identify a possible mechanical power threshold for injury, we first divided the whole population into deciles of mechanical power. As shown in Fig. 2, alveolar ruptures, inflammation, alveolar edema and atelectasis increased markedly after the 5th decile of MP_ratio_, i.e., MP_ratio_ > 4.46 (rounded to 4.5).

To confirm the graphical results, we performed a k-means clustering analysis of all the histological variables. This analysis identified two distinct injury clusters, as shown in Figure E1. The first cluster had significantly higher MP_ratio_ and higher lung histological scores, with greater alveolar edema and intra-alveolar hemorrhage, atelectasis, inflammation, septal ruptures and vascular congestion (alveolar damage group). The second cluster, which was exposed to lower MP_ratio_, predominantly exhibited higher perivascular edema and intravascular thrombi (vascular damage group). The assignment to either of these histological clusters was associated to the MP_ratio_ (*p* < 0.001, Table E1). Logistic regression identified an MP_ratio_ > 4.5 as a discriminant value between the two clusters, which aligns with the upper limit value of the fifth decile of MP_ratio_ (see Table E2). Consequently, we divided the population into low (≤ 4.5) and high (> 4.5) MP_ratio_ groups according to this threshold.

## Results

### Pathological findings

**Fig. 3:**
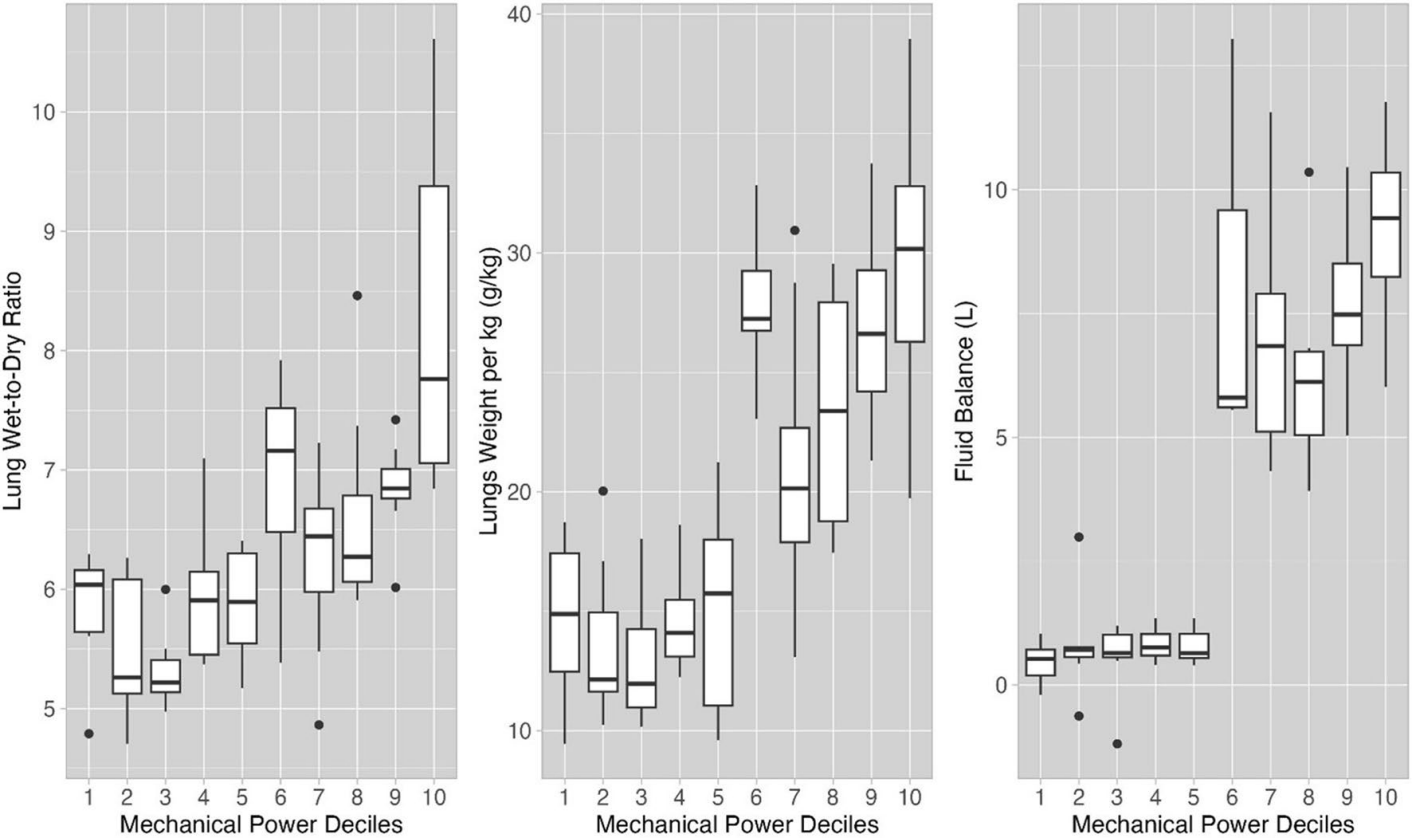
Lung edema and mechanical power ratio. Lung wet-to-dry ratio (**A**), lung weight (kg^−1^) (**B**) and fluid balance (**C**) as a function of the MP_ratio_ deciles. The MP_ratio_ limits of each decile are the same as in Fig. 1. Upon visual inspection, at MP_ratio_ greater than 4.5, lung wet-to-dry ratio, lung weight and fluid balance also markedly increased

In Figs. 2 and 3, we report the gross pathological findings measured in the MP_ratio_ deciles. Notably, the lung weight in the MP_ratio_ group higher than threshold of 4.5 was 68% greater compared to the lower MP_ratio_ group (Fig. 3A). A similar relationship was observed for the wet-to-dry ratio (Fig. 3B), and the fluid balance (Fig. 3C). Fluid balance was associated to the variations in lung weight (*R*^2^ = 0.61, *p* < 0.001, Figure E2, panel A) and histological VILI score (*R*^2^ = 0.32, *p* < 0.001, Figure E2, panel B, Table E3). The complete set of histological findings, including intra-alveolar hemorrhage, vascular congestion, perivascular edema, and intravascular thrombi, is reported in Table 2 as well as lungs’ weight and wet-to-dry ratio of lungs and other organs.

**Table 2:** Histological and pathological findings

Groups Pigs number	Low MPratio (≤ 4.5) *n* = 41	High MPratio (> 4.5) *n* = 41	*p *value
Alveolar ruptures	40 [40–42]	58 [49.5–94.5]	< 0.001
Inflammation	100 [62–134]	228 [204–281]	< 0.001
Alveolar edema	38 [8–46]	130 [58–196]	< 0.001
Atelectasis	4 [0–36]	50 [37–65]	< 0.001
Intra-alveolar hemorrhage	40 [38–52]	112 [78–174]	< 0.001
Vascular congestion	80 [67–106]	242 [194–304]	< 0.001
Perivascular edema	40 [0–56]	0 [0–0]	< 0.001
Intravascular thrombi	4 [0–44]	0 [0–0]	< 0.001
Histological VILI score	376 [327–477]	884 [666–1000]	< 0.001
Lung weight (g kg^−1^)	14.1 [12.1–20.0]	20.8 [15.7–27.7]	< 0.001
Wet-to-dry lung	5.64 [5.25–6.15]	6.80 [6.35–7.38]	< 0.001
Wet-to-dry liver	4.61 [4.40–4.84]	4.02 [3.84–4.22]	< 0.001
Wet-to-dry kidney	5.67 [5.43–6.17]	5.52 [4.53–6.04]	0.047
Wet-to-dry muscle	3.92 [3.62–4.15]	3.64 [3.19–4.07]	0.022
Wet-to-dry bowel	5.28 [5.00–5.77]	5.59 [5.22–5.95]	0.055

*MP*_*ratio*_ mechanical power ratio, *VILI* ventilator-induced lung injury

Histological and anatomic variables in low and high mechanical power ratio groups. Values are expressed as median [IQR]. Student’s *T* test or Wilcoxon–Mann–Whitney *U* test, as appropriate

*p* < *0.05* was considered significant

### Physiological variables in high and low MP_ratio_ group at 0.5 h after the application of the experimental protocol

As shown in Table 3, ventilatory settings changed significantly from baseline after the application of the experimental protocol. Specifically, due to the protocol, minute ventilation, PEEP, mean airway pressure, plateau pressure, were notably higher in the high MP_ratio_ group compared to the low MP_ratio_ group. Similarly, gas exchange, respiratory mechanics and hemodynamic variables were different in the two groups, immediately after the implementation of the experimental protocol. Indeed, at 0.5 h, PaCO_2_ was lower and PaO_2_/FiO_2_ ratio was higher in the high MP_ratio_ group, while respiratory system elastance and lung stress were significantly higher in high MP_ratio_ group; as were most of hemodynamic variables. A summary of all respiratory mechanics, gas exchange and hemodynamic variables is presented in Table E3.

**Table 3:** Physiological variables after change of mechanical power

	Low MPratio (≤ 4.5) *n* = 41	High MPratio (> 4.5) *n* = 41	*p*_GROUP_	*p*_TIME_	*p*_INTER_
Mechanical power (J min^−1^ kg^−1^)					
0 h	0.21 [0.20–0.23]	0.21 [0.18–0.23]	< 0.001	0.003	< 0.001
0.5 h	0.29 [0.27–0.33]†	0.80 [0.56–1.08]*†			
Minute ventilation (L min^−1^ kg^−1^)					
0 h	0.18 [0.17–0.19]	0.18 [0.17–0.21]	< 0.001	< 0.001	< 0.001
0.5 h	0.18 [0.16–0.20]	0.37 [0.24–0.49]*†			
PaO_2_/FiO_2_ (mmHg)					
0 h	525 [498–552]	546 [534–572]*	< 0.001	0.307	< 0.001
0.5 h	512 [432–548]	602 [564–628]*†			
PaCO_2_ (mmHg)					
0 h	45.5 [42.0–48.8]	46.0 [42.8–48.2]	0.003	< 0.001	0.001
0.5 h	38.0 [33.2–41.8]†	29.0 [19.8–35.2]*†			
Physiological dead space (%)					
0 h	53 [48–57]	50 [45–54]*	< 0.001	< 0.001	0.611
0.5 h	48 [45–52]†	44 [37–49]*†			
Respiratory system elastance (cmH_2_O L^−1^)					
0 h	30.1 [27.2–35.2]	38.0 [35.3–43.5]*	< 0.001	0.011	< 0.001
0.5 h	29.5 [25.1–35.0]	36.1 [31.8–72.4]*†			
Stress (cmH_2_O)					
0 h	7.0 [6.0–8.1]	6.8 [6.0–7.9]	< 0.001	< 0.001	< 0.001
0.5 h	10.4 [7.6–12.9]†	15.7 [10.5–29.5]*†			
Central venous pressure (mmHg)					
0 h	2 [0–4]	7 [5–9]*	< 0.001	< 0.001	< 0.001
0.5 h	2 [1–3]	10 [7–12]*†			
Mean pulmonary artery pressure (mmHg)					
0 h	18 [15–21]	20 [18–22]*	0.001	< 0.001	0.095
0.5 h	19 [17–26]†	24 [20–32]*†			
Cardiac output (L min^−1^ kg^−1^)					
0 h	0.10 [0.09–0.11]	0.16 [0.15–0.19]*	< 0.001	< 0.001	< 0.001
0.5 h	0.10 [0.09–0.12]	0.19 [0.16–0.22]*†			

Ventilatory setting, gas exchange, respiratory mechanics and hemodynamic variables at baseline (0 h) and after 30 min from the beginning of the experimental phase (0.5 h) according to MP_ratio_ groups. A two-way ANOVA was applied to investigate the difference in time-course in both groups

*p < 0.05 vs Low MP_ratio_; †*p* < 0.05 vs 0 h

### Physiological variables during time-course of the experiment

**Fig. 4:**
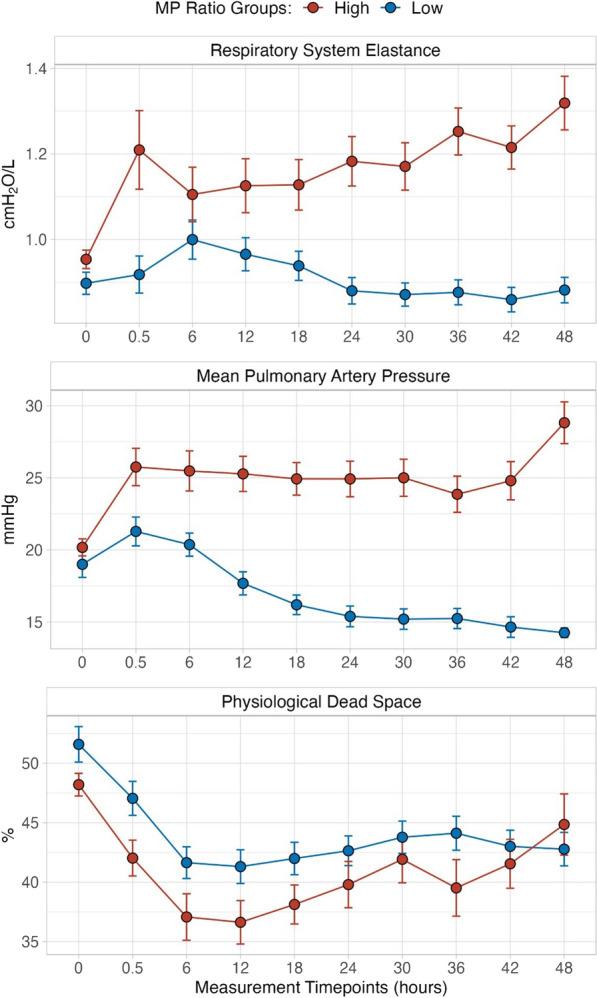
Time-course of normalized respiratory system elastance (**A**), mean pulmonary arterial pressure (**B**) and physiological dead space (**C**), in the high (red circles) and low (blue circles) MP_ratio_ groups. At the two-way ANOVA analysis: Respiratory system elastance: *p*_GROUP_ < 0.001, *p*_TIME_ = 0.039, and *p*_INTERACTION_ < 0.001. Mean pulmonary artery pressure: *p*_GROUP_ = 0.003, *p*_*TIME*_ < 0.001, and *p*_INTERACTION_ < 0.001. Physiological dead space: *p*_*GROUP*_ = 0.550, *p*_*TIME*_ = 0.097, and *p*_INTERACTION_ = 0.039. See also Table E4 for the complete sets of variables

In Fig. 4, we display the time-course of three representative variables related to respiratory mechanics (respiratory system elastance, panel A), hemodynamics (mean pulmonary artery pressure, panel B) and gas exchange (physiological dead space, panel C). As shown, once the experimental phase began, all variables significantly and progressively deteriorated in the high MP_ratio_ group over the duration of the experiment. A summary of the time-course change of the most relevant pathophysiological variables is presented in Table 4. Of note, ventilatory settings (i.e., tidal volume, respiratory rate, PEEP and I:E ratio at 0.5 h) were maintained constant at the same values reported in Table 3. However, during the experiment, significant changes were observed in plateau pressure and MP, both of which increased to a greater extent in the high MP_ratio_ group. In addition, PaO_2_/FiO_2_, PaCO_2_ and physiological dead space significantly worsened in the high MP_ratio_ group; the same behavior was observed for the lung mechanics and for all hemodynamic variables. A complete summary of the variables is reported in Table E4.

**Table 4:** Physiological variables time-course

	Low MPratio (≤ 4.5) *n* = 41	High MPratio (> 4.5) *n* = 41	*p*_GROUP_	*p*_TIME_	*p*_INTER_
Mechanical power (J min^−1^ kg^−1^)					
0.5 h	0.29 [0.25–0.33]	0.82 [0.56–1.10]*	< 0.001	0.051	0.002
48 h	0.29 [0.23–0.33]	0.86 [0.62–1.16]*			
Minute ventilation (L min^−1^)					
0.5 h	4.98 [4.52–5.70]	8.20 [5.64–11.80]*	< 0.001	0.287	0.061
48 h	4.94 [4.53–5.71]	8.22 [5.27–11.70]*			
PaO_2_/FiO_2_ (mmHg)					
0.5 h	512 [412–540]	595 [548–622]*	< 0.001	< 0.001	< 0.001
48 h	505 [459–526]	545 [509–576]*†			
PaCO_2_ (mmHg)					
0.5 h	40 [36–56]	30 [22–36]*	< 0.001	< 0.001	< 0.001
48 h	33 [29–43]†	25 [16–30]*†			
Physiological dead space					
0.5 h	48 [45–52]	43 [36–49]*	0.571	< 0.001	0.015
48 h	43 [39–47]†	48 [32–55]			
Respiratory system elastance (cmH_2_O L^−1^)					
0.5 h	29.5 [24.7–35.0]	36.1 [31.7–71.9]*	< 0.001	0.039	< 0.001
48 h	28.6 [25.2–33.7]	46.6 [34.4–66.4]*			
Stress (cmH_2_O)					
0.5 h	10.4 [7.67–13.0]	16.4 [10.7–31.7]*	< 0.001	0.401	0.002
48 h	9.8 [6.75–13.0	21.4 [16.7–27.5]*			
Central venous pressure (mmHg)					
0.5 h	2 [1–3]	8 [5–11]*	< 0.001	< 0.001	0.006
48 h	2 [1–3]	10 [7–14]*			
Mean pulmonary arterial pressure (mmHg)					
0.5 h	18 [17–25]	24 [20–32]*	< 0.001	< 0.001	< 0.001
48 h	14 [13–15]†	31 [21–35]*			
Cardiac output (L min^−1^ kg^−1^)					
0.5 h	0.10 [0.09–0.12]	0.19 [0.16–0.22]*	< 0.001	< 0.001	< 0.001
48 h	0.08 [0.07–0.09]†	0.14 [0.11–0.20]*†			

Values are expressed as median [IQR]. Time-course of physiological variables from 0.5 to 48 h of experimental mechanical power ratio application in high and low mechanical power ratio groups. See Table E3 for the complete set of variables. Two-way ANOVA analysis: *p*_GROUP_ refers to difference between groups; *p*_TIME_ refers to change with time; *p*_INTER_ refers to different patterns of variables change over time in the two groups. Post hoc by pairwise Student’s *T* test

*p < 0.05 low vs high mechanical power ratio groups; †*p* < 0.05 0.5 vs 48 h

## Discussion

This experimental analysis investigates the role of mechanical power (MP) in generating VILI in large, healthy animals. Given the absence of pre-existing lung disease (healthy baseline), the progression observed over time can be attributed to mechanical ventilation, implying the development of VILI. This study has identified a specific power threshold that results in histologically confirmed lung injury.

The main results show that, with an applied MP ranging from 2.6 to 63.4 J min^−1^ (0.1–2.3 J min^−1^ kg-1), injury occurred when the applied MP_ratio_ exceeded approximately 4.5 times its normal expected value. This normal MP value was computed using data from De Robertis et al. [14] for domestic pigs, similar in weight to our population. The median value of normal expected MP was 0.094 J min^−1^ kg^−1^, close to the value expected in normal human (i.e., 6–7 J min^−1^ in a 70 kg individual) [15].

The two groups of animals defined as having a lower or higher MP_ratio_ than threshold of 4.5 differed considerably in terms of respiratory and hemodynamic physiology during the experiment. These differences were also associated with the higher fluid balance needed to maintain the hemodynamics in the group at higher MP_ratio_ [16]. Over time, from 0.5 to 48 h, key marker variables diverged between the two groups (Table E4) with the high MP_ratio_ group showing increased physiological dead space and worsened respiratory mechanics. These data are consistent with the appearance and worsening of structural lung lesions in that cohort. Despite increased histologically identified atelectasis, venous admixture decreased in the high MP_ratio_ group. This is not surprising, considering that atelectasis due to VILI is easily recruitable during inspiratory phase [17] and that oxygenation variables are not closely related to VILI-related outcome, as shown in the ARMA trial [6].

**Fig. 5:**
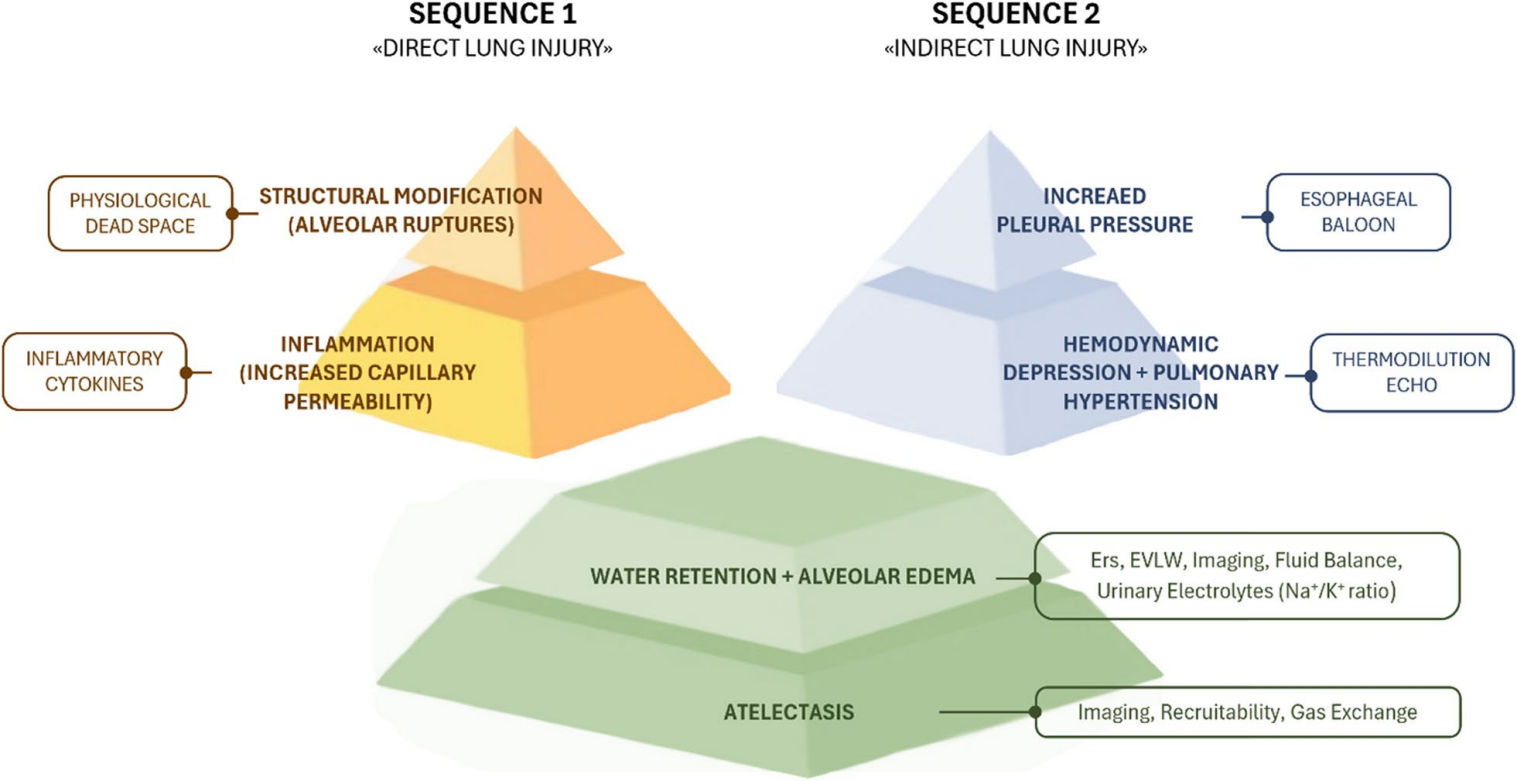
Possible sequences leading to ventilator-induced lung injury in healthy lung. Sequence 1, the intensity of mechanical ventilation is such as to induce structural anatomical modifications, which in turn lead to inflammatory reaction. The physiological variables in theory more associated with these alterations should be the physiological dead space and the inflammatory cytokines, both not specific for lung injury. Sequence 2: the intensity of mechanical ventilation is not such as to induce anatomical damage, but sufficient to interfere with normal hemodynamics. In theory, the first event is the increased intrathoracic pressure followed by all the hemodynamic consequences. The estimate of change of intrathoracic pressure requires the esophageal balloon, while several methods are available to assess the hemodynamic status. Both “direct” and “indirect” lung injury sequences convey in the same final pattern, characterized by water retention, alveolar edema and atelectasis. In the figure we report the common physiological variables which should be more associate with these events

This study highlights the ambiguity in defining VILI, as it remains unclear which variables (anatomic, hemodynamics, mechanics, and gas exchange) are direct expressions of VILI or merely associated damage co-factors. Indeed, some authors have included the hemodynamic alterations and fluid balance under the VILI umbrella, while some others have not [18, 19]. Indeed, the use of terms as ventilator-induced lung injury or ventilation-induced lung injury or ventilator-associated lung injury reflect the lack of clarity of this concept. A possible model for VILI development during mechanical ventilation in healthy lungs [20] may consist of two sequences (Fig. 5).

### Sequence 1: structural alterations

The gold standard for VILI should be the alveolar ruptures and extracellular matrix fragmentation due to the excessive MP, leading to increased dead space and lung elastance [20], as demonstrated in this study in the high MP_ratio_ group. *Inflammation*: alveolar ruptures and extracellular matrix fragmentation led to marked inflammation, with increased capillary permeability. This phenomenon may be tracked by changes in the level of inflammatory cytokines [21], and by a marked increase of inflammation as observed in histological samples of high MP_ratio_ group. Water retention/pulmonary hypertension/pulmonary edema/increased lung weight: Several variables, are associated with these phenomena. Water retention may be estimated by fluid balance, which was clearly more positive in the high MP_ratio_ group than in the low one. Lung weight (suggesting lung edema) was also markedly greater in the high MP_ratio_ group and was significantly associated with the fluid balance and with structural histological changes of the lung. It appears, therefore, that the formation of lung edema depends on both functional and structural changes of lung parenchyma. *Compression atelectasis*: lung edema and increased lung weight inevitably lead to compression atelectasis [22, 23]. The best way to measure it is the assessment of recruitability, as only atelectatic (i.e., compressed but empty) pulmonary units may be recruited. Unfortunately, we did not measure recruitability in our experiments. However, the frequency of atelectasis detected by histology was significantly higher in the high MP_ratio_ group. The consequent atelectrauma possibly contributed to enhance the inflammatory reaction and its consequences.

### Sequence 2

It is possible that “harmful mechanical ventilation”, although not so severe as to induce structural changes, is sufficient to induce a marked hemodynamic response. Indeed, in our study we found that mechanical ventilation may be associated with increased pulmonary artery pressure. To support hemodynamics in the high MP_ratio_ group, fluid balance was markedly higher, with consequences similar to what is described in sequence 1. In essence, the difference between the two sequences is the lack of structural alteration, which selectively characterizes sequence 1. In our study, we had clear signs of hemodynamic compromise which appear more than a threshold-linked phenomenon, as they appeared proportional to the MP.

### Limitations

Using healthy animals to study ventilator-induced lung injury (VILI) might be seen as a limitation. However, in our research, we consistently use healthy subjects to ensure that the only variable affecting lung damage is the mechanical ventilation per se, free from the confounding factors found in the “ARDS models”. In addition, over the course of our experimental model factors like inhomogeneity and stress raisers develop progressively, aligning the model more closely with the temporal evolution of the ARDS pathophysiology.

This study presents several other limitations primarily due to the retrospective design, which includes data from several experiments conducted at different times. The data derived from three distinct studies, each with potentially different use of fluid and vasopressors. This heterogeneity might have affected the results of the cluster analysis, potentially categorizing animals into groups based on the conditions of one larger study that employed significantly higher mechanical power and fluid balance. Additionally, the distribution of mechanical power ratio (MP_ratio_) may have been more discrete than continuous due to the nature of the experimental design.

Another possible limitation may be the lack of comparisons of MP ratio to other possible “VILI markers” as driving pressure and the index proposed by Costa (4DP + RR) in which DP is the driving pressure and RR is the respiratory rate [24]. However, both driving pressure and frequency are included in the mechanical power equation and therefore these express the same elements of VILI. The direct comparison between mechanical power and driving pressure or 4DP + RR may be the focus of a different study but this may be inappropriate in an experimental model as the specific weighting of DP and RR may be different from the 4:1 found in human clinical trials.

However, we think that despite these limitations the results are valid. Variations in the expected MP do not compromise the validity of identifying a VILI threshold. The distribution of MP and MP_ratio_ seems to be near-continuous and well distributed across the range of MPratio (see Figure E4), and the fluid overload was the consequence of the high MP, rather than the cause.

### The challenges of assessing VILI

These results and limitations reflect a longstanding debate in the understanding of VILI, which was already raised in the early descriptions of ARDS. Should the physiological hemodynamic response and its consequences (e.g., positive fluid balance and water retention due to the kidney response) be considered VILI in the absence of structural alterations?

#### Healthy lung

We argue that if one starts with healthy animals, whatever alterations we found were likely due to mechanical ventilation itself, as no prior inflammation, pulmonary hypertension or edema were present prior to its initiation. Accordingly, water retention, fluid balance and hemodynamic compromise should be considered part of VILI. Perhaps, this condition is better described by a term such as *ventilator-induced injury*, as most vital extrapulmonary organ systems, are indirectly compromised by mechanical ventilation.

#### Diseased lung

While the bulk of our results strongly suggest that a distinct MP_ratio_ greater than 4.5 causes VILI to become evident, this threshold cannot be, as such, translated to diseased lung and to human ARDS. Indeed, in diseased lung the damage related to the energy input should ideally be normalized to the size of the ventilatable lung, i.e., to the size of baby lung. While inflating the respiratory system requires a level of energy related to its mechanical characteristics, the capacity of the baby lung to receive it is less. The specific energy delivered per lung unit is an inverse function of baby lung size.

## Conclusion

Our results suggest the existence of an MP_ratio_ threshold in healthy lungs.

Beyond this threshold, structural and functional changes consistent with Ventilator-Induced Lung Injury (VILI) occur, as indicated by the convergence of histological, physiological, and anatomical alterations. In lungs that are already injured, this threshold is likely to be different. This is due to the application of potentially damaging energy to a smaller aerated lung that is more vulnerable to strain. These findings contribute towards a correct understanding of the issue: distinguishing between the natural progression of the disease and the adverse impact of mechanical ventilation.

## Supplementary Information

Supplementary Material 1.

## Data Availability

The datasets used and/or analyzed during the current study are available from the corresponding authors on reasonable request.
