# Correction: Mechanical power ratio threshold for ventilator-induced lung injury

**DOI:** 10.1186/s40635-024-00666-z

**Published:** 2024-09-12

**Authors:** Rosanna D’Albo, Tommaso Pozzi, Rosmery V. Nicolardi, Mauro Galizia, Giulia Catozzi, Valentina Ghidoni, Beatrice Donati, Federica Romitti, Peter Herrmann, Mattia Busana, Simone Gattarello, Francesca Collino, Aurelio Sonzogni, Luigi Camporota, John J. Marini, Onnen Moerer, Konrad Meissner, Luciano Gattinoni

**Affiliations:** 1 Department of Anesthesiology, University Medical Center Göttingen, Göttingen, Germany; 2 Department of Medical and Surgical Sciences, Alma Mater Studiorum, University of Bologna, Bologna, Italy; 3 Department of Health Sciences, University of Milan, Milan, Italy; 4 IRCCS San Raffaele Scientific Institute, Milan, Italy; 5 Department of Health Sciences, Section of Anesthesiology, Intensive Care and Pain Medicine, University of Florence, Florence, Italy; 6 Department of Anesthesia, Intensive Care and Emergency, “City of Health and Science” Hospital, Turin, Italy; 7 Department of Pathology, ASST Bergamo Est, Seriate, Italy; 8 Department of Adult Critical Care, Health Centre for Human and Applied Physiological Sciences, Guy’s and St. Thomas’ NHS Foundation Trust, London, UK; 9 Department of Pulmonary and Critical Care Medicine, University of Minnesota and Regions Hospital, St. Paul, MN USA

**Correction: Intensive Care Medicine Experimental (2024) 12:65** 10.1186/s40635-024-00649-0

Following publication of the original article, the following two concerns were brought to the attention of the journal: the author affiliations were incorrectly detailed in the PDF version of the article; in Additional file 1, the paragraph regarding the expected mechanical power formula was missing. The published article [1] has since been corrected to address these issues.
