# Supplementary material for: Mechanical power ratio threshold for ventilator-induced lung injury
Source: Intensive Care Med Exp. 2024 Jul 30;12:65. doi: 10.1186/s40635-024-00649-0 (PMC11289208; doi:10.1186/s40635-024-00649-0)
Supplement: Supplementary file 1 — Supplementary Material 1. [file 40635_2024_649_MOESM1_ESM.docx]

**Mechanical power ratio threshold for ventilator induced lung injury:**

**Online Supplement**

Rosanna D’Albo*, Tommaso Pozzi*, Rosmery V. Nicolardi, Mauro Galizia, Giulia Catozzi, Valentina Ghidoni, Beatrice Donati, Federica Romitti, Peter Herrmann^,^ Mattia Busana, Simone Gattarello, Francesca Collino, Aurelio Sonzogni, Luigi Camporota, John J. Marini, Onnen Moerer, Konrad Meissner, Luciano Gattinoni

* The authors contributed equally to this work.

**Additional Material and Methods**

***Study population***

Our experimental study originally consisted in 82 female domestic pigs. These piglets (body weight 26.6 ± 3.3 kg) were enrolled in previous experiments (Vassalli *et al.*, Romitti *et al.* and Busana *et al.*, ref 10-11-12 of the main manuscript), and were ventilated in volume-controlled mechanical ventilation with mechanical power (MP) values, ranging from 2.60 to 63.4 J min^-1^ for 48 hours, in their natural prone position. All experiments were approved by LAVES (Niedersächsisches Landesamt für Verbraucherschutz und Lebensmittelsicherheit, Projekte 18/2795, 19/3141) and they were carried out in a dedicated operating room in the animal facility within Göttingen Medicine University (UMG), Göttingen, Germany.

***Animal preparation***

Before entering the operating room, premedication was managed by veterinarian with anxiolytic drugs (Ketamine 10 mg kg^-1^, Azaperone 2 mg kg^-1^) administered by intra-muscular injection into the neck. Subsequently, a peripheral venous access was placed at the auricular vein, and 20 mg of propofol was administered. During the experiment the animals were kept under intravenous general anesthesia with Propofol, Sufentanyl and Midazolam. Before the experimental phase the animals were instrumented with the following devices:

- Endotracheal tube (size 6.5 -7 mm)
- Urinary catheter (5Fr)
- Adult esophageal catheter (8 Fr) Nutrivent with esophageal balloon; the correct positioning of the esophageal catheter was checked by an end-expiratory occlusion test
- Central venous catheter (5 Fr) in the jugular vein, ultrasound-guided
- Pulmonary artery catheter Swan-Ganz (5 Fr) through an introducer (7 Fr) in the jugular vein, ultrasound guided
- Arterial PiCCO catheter (5 Fr) in the femoral artery, ultrasound guided

***Physiological variables***

At each timepoint, we measured: respiratory mechanics, hemodynamics and gas exchange variables.

The derived physiological variables were measured according to the formulae reported;

$$Respiratory System Elastance (E_{rs}, {cmH}_{2}O\cdot L^{-1})=\frac{{Paw}_{plat}-PEEP}{V_{T}}$$

$$Normalized respiratory system elastance (E_{rs}, {cmH}_{2}O\cdot kg\cdot{mL}^{-1}) = \frac{Ers \cdot kg}{1000}$$

$$Chest Wall Elastance {(E}_{rs}, {cmH}_{2}O\cdot L^{-1})=\frac{{Pes}_{plat}-{Pes}_{ee}}{V_{T}}$$

Where Paw_plat_ is plateau airway pressure, PEEP is the end-expiratory airway pressure, Pes_plat_ is plateau esophageal pressure measured at pleateau, Pes_ee_ is esophageal pressure measured at PEEP, all expressed in cmH_2_O and V_T_ is tidal volume expressed in L.

$$Lung Elastance \left( EL , {cmH}_{2}O\cdot L^{-1} \right)= Ers- Ecw$$

Lung stress was calculated as follows:

$$Lung stress={Paw}_{plat}\cdot\frac{EL}{Ers}$$

Venous admixture fraction was calculated:

$$Venous admixture fraction =\frac{{CcO}_{2}-{CaO}_{2}}{{CcO}_{2}-{CvO}_{2}}$$

Where CaO2 is arterial oxygen content, CvO2 is venous oxygen content and CcO2 is capillary content oxygen and were calculated as follows: (reference)

$$CaO_{2}= 1.39\left( \frac{ml}{gr} \right)\cdot Hb\left( \frac{g}{dl} \right)\cdot SaO_{2}(\%) + P_{a}O_{2}\left( mmHg \right)\cdot0.0031(ml {mmHg}^{-1})$$

$CvO_{2}= 1.39\left( \frac{ml}{gr} \right)\cdot Hb\left( \frac{g}{dl} \right)$ $\cdot SvO_{2} (\%)$+$P_{v}O_{2}\left( mmHg \right)\cdot0.0031(ml {mmHg}^{-1})$

$CcO_{2}= 1.39\left( \frac{ml}{gr} \right)\cdot Hb\left( \frac{g}{dl} \right)$ + ${P_{A}O}_{2}\left( mmHg \right)\cdot0.0031(ml {mmHg}^{-1})$

Where Hb is hemoglobin, P_A_ is alveolar pressure and was calculated as follow:

$${P_{A}O}_{2}=\left( 760-47 \right) mmHg\times F_{i}O_{2}-\frac{P_{a}{CO}_{2}}{0.8}$$

Where FiO2 is fraction of inspired oxygen and 0.8 is respiratory quotient.

Physiological dead space was calculated as:

$$Physiological dead space =\frac{Pa{CO}_{2}-PE{CO}_{2}}{Pa{CO}_{2}}\cdot100$$

Where PaCO2 is arterial partial pressure of carbon dioxide and PECO2 is mixed expired carbon dioxide tension.

The Functional Residual Capacity (FRC) was measured by the helium dilution technique:

$$Functional Residual Capacity \left( mL \right)=\frac{Vi * (Ci - Cf)}{Cf}$$

Where Vi is the mixing volume of Helium (1 Liter), Ci is the initial concentration of the helium in the volume Vi (14 %) and Cf is the final concentration after 10 mixed in the closed circuit (V_T_+FRC).

*Expected mechanical power*

The expected mechanical power was computed according the following formula:

$$MP =0.098\times RR\times\{{V_{T}}^{2}\times\left[ \frac{1}{2}\times{EL}_{rs}+RR\times\frac{1+I:E}{60\times I:E}\times R_{aw} \right]+V_{T}\times PEEP\}$$

Where MP is the mechanical power, RR is the respiratory rate, V_T_ is the tidal volume, EL_rs_ is the respiratory system elastance, I:E is the inspiratory/expiratory ratio, R_aw_ is the airways resistance and PEEP is the positive end-expiratory pressure. To compute the expected mechanical power in the above formula the following physiological normal values were inserted according to De Robertis E et al: Elastic properties of the lung and the chest wall in young and adult healthy pigs. *Eur Respir J* 2001; 17: 703-711:

| **Respiratory Rate** *(breath∙min^-1^)* | 20 |
| --- | --- |
| **Tidal Volume** *(mL*∙*kg^-1^)* | 10 |
| **Respiratory System Elastance**  *(cmH_2_O∙mL^-1^ kg* *^1^ )* | 0.75 |
| **I:E** | 0.5 |
| **Airways resistance** *(cmH_2_O∙L^-1^ ∙s^-1^ kg* *^1^)* | 3.9 |
| **PEEP** *(cmH_2_O)* | 0 |

**Additional Results**

**Figure E1.** Cluster analysis. BI-dimensional plot for visualization of the cluster analysis obtained by the k-means algorithm on the x and y axis, the two dimensions of the rendering with the percentage of variance explained by each dimension in brackets.


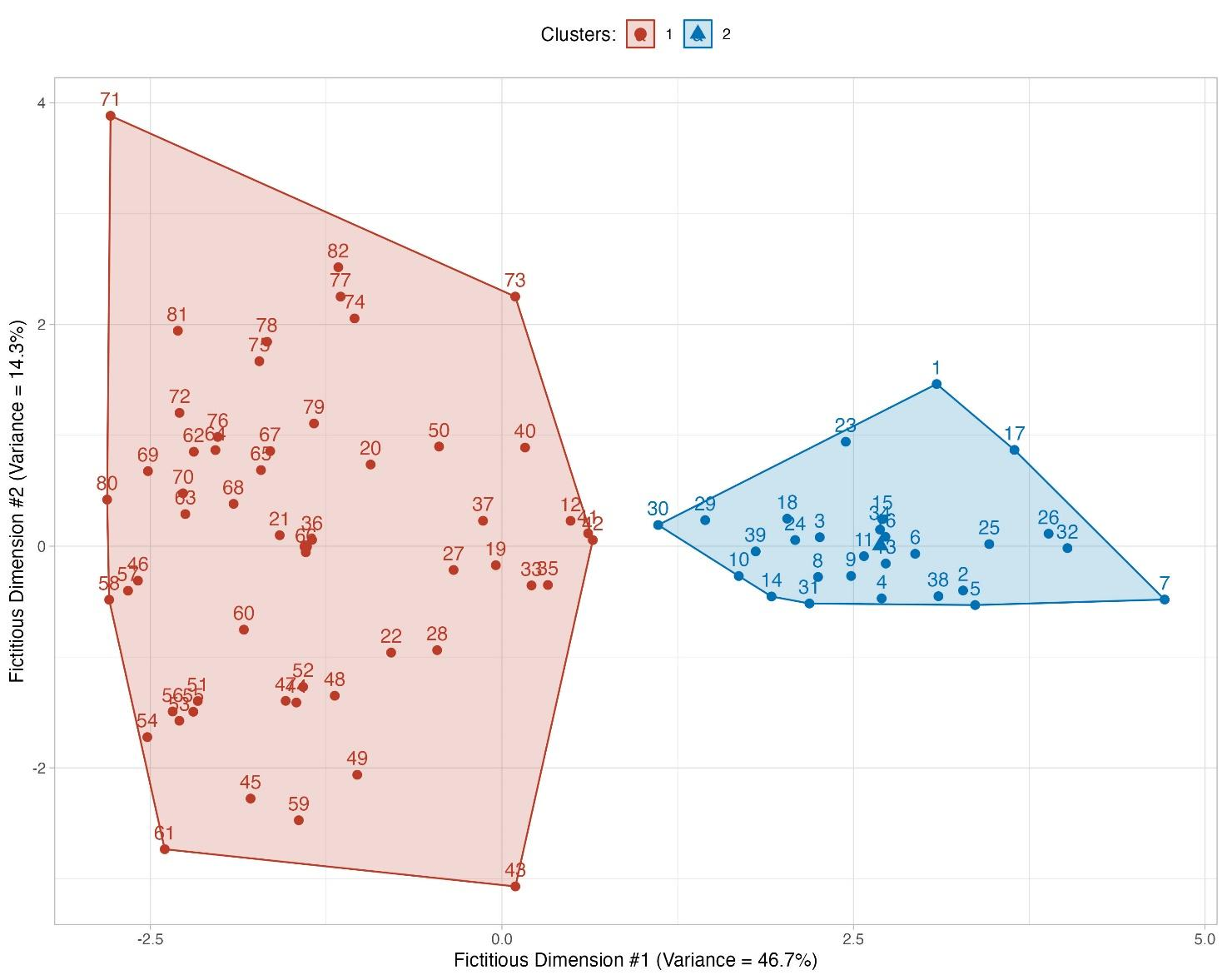


**Figure E2.** Lung weight (Panel A) and histological VILI score (Panel B) as a function of fluid balance.

*
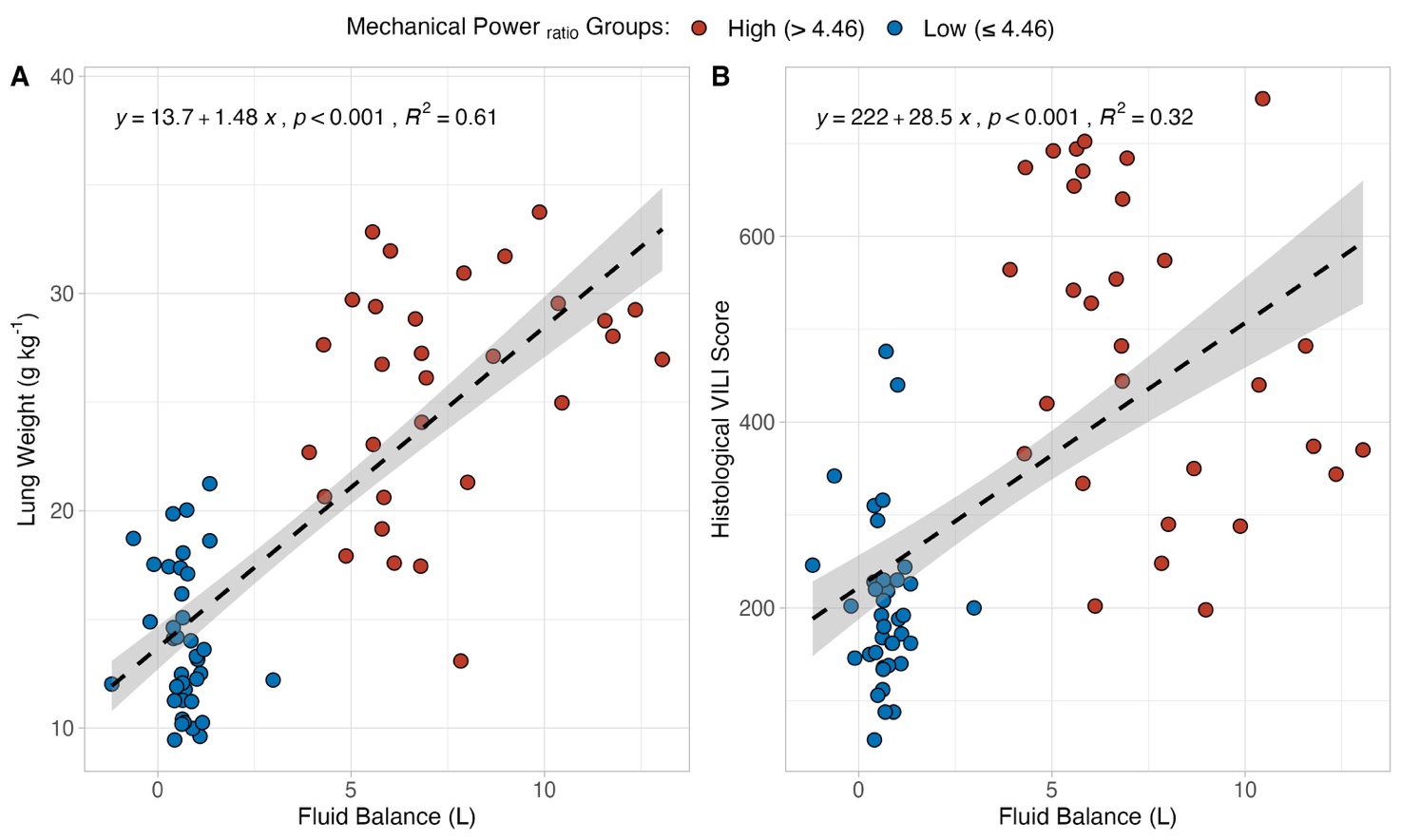
*

**Figure E3.** Distribution of MP (left) and MPratio (right) in the study population according to deciles

of MPratio.

**Table E1**. *Details of the logistic regression and the logistic-based model for Youden Index calculation:*

| **Predictor** | ***p*** | **AIC** | **Accuracy** | **AUC** | **Youden Index** | **Best Cutoff Value** |
| --- | --- | --- | --- | --- | --- | --- |
| *Mechanical Power Ratio* | < 0.001 | 95.5 | 0.829 | 0.836 | 0.740 | 4.46057 |

**Table E2.** *Details of the multivariate regression model with lung weight as dependent variable and histological VILI score and fluid balance as predictors.*

| **Predictor** | **Slope** | ***p*** | **R^2^** |
| --- | --- | --- | --- |
| Histological VILI Score | $1.06\times{10}^{-2}$ | *0.002* | 0.65 |
| Fluid Balance | $1.18\times{10}^{-3}$ | *<0.001* |  |

***Table E3***. *Physiological variables at baseline and 0.5 hours after protocol initiation.*

| **Groups**  *Pigs Number* | **Low** *MP_ratio_ (≤ 4.5)*  *n = 41* | **High** *MP_ratio_ (> 4.5)*  *n = 41* | ***p_GROUP_*** | ***p_TIME_*** | ***p_INTER_*** |
| --- | --- | --- | --- | --- | --- |
|  | | | | | |
| **Mechanical Power (*J*∙*min^-1^*∙*kg^-1^*)**  0 h  0.5 h | 0.21 [0.20-0.23]  0.29 [0.27-0.33]† | 0.21 [0.18-0.23]  0.80 [0.56-1.08]*† | *<0.001* | *0.003* | *<0.001* |
| **Minute Ventilation (*L*∙*min^-1^*∙*kg^-1^*)**  0 h  0.5 h | 0.18 [0.17-0.19]  0.18 [0.16-0.20] | 0.18 [0.17-0.21]*  0.37 [0.24-0.49]*† | *<0.001* | *<0.001* | *<0.001* |
| **Tidal Volume *(mL*∙*kg^-1^)***  0 h  0.5 h | 9.6 [9.3-9.8]  15.7 [9.8-19.6] | 9.5 [8.8-10.4]*  14.2 [12.5-30.6]*† | *0.005* | *<0.001* | *0.004* |
| **Respiratory Rate (*breath∙min^-1^*)**  0 h  0.5 h | 18 [18-21]  10 [8-21]† | 20 [18-22]  16 [11-40]* | *<0.001* | *0.192* | *<0.001* |
| **Plateau Pressure *(cmH_2_O)***  0 h  0.5 h | 12.1 [11.0-13.6]  17.4 [13.9-20.8]† | 14.2 [13.2-14.9]*  29.9 [21.2-45.3]*† | *<0.001* | *<0.001* | *<0.001* |
| **Positive End-Expiratory Pressure *(cmH_2_O)***  0 h  0.5 h | 4 [4-4]  4 [4-6] | 5 [5-5]*  5 [5-25]*† | *<0.001* | *<0.001* | *<0.001* |
| **Driving pressure *(cmH_2_O)***  0 h  0.5 h | 8.2 [7.7-9.4]  13.0 [9.5-15.5]† | 9.0 [8.0-10.3]  23.0 [11.9-27.4]*† | *<0.001* | *<0.001* | *<0.001* |
| **Mean Airway Pressure *(cmH_2_O)***  0 h  0.5 h | 8 [7-8]  9 [8-11]† | 9 [9-10]*  16 [12-30]*† | *<0.001* | *<0.001* | *<0.001* |
|  | | | | | |
| **PaO_2_/FiO_2_ (*mmHg*)**  0 h  0.5 h | 525 [498-552]  512 [432-548] | 546 [534-572]*  602 [564-628]*† | *<0.001* | *0.307* | *<0.001* |
| **PaCO_2_ (*mmHg*)**  0 h  0.5 h | 45.8 [42,48.8]  37.5 [33,42]† | 49 [42.8,48]  29.3 [19.5,35.3]*† | *0.003* | *<0.001* | *0.001* |
| **Physiological Dead Space (%)**  0 h  0.5 h | 53 [48-57]  48 [45-52]† | 50 [45-54]  44 [37-49] | *<0.001* | *<0.001* | *0.611* |
| **Venous Admixture (%)**  0 h  0.5 h | 14.2 [11.7-18.0]  16.8 [14.6-20.7]† | 7.59 [1.95-15.70]*  8.80 [2.51-14.80]* | *<0.001* | *0.043* | *0.018* |
|  | | | | | |
| **Respiratory System Elastance (*cmH_2_O∙L^-1^*)**  0 h  0.5 h | 30.1 [27.2-35.2]  29.5 [25.1-35.0] | 38.0 [35.3-43.5]*  36.1 [31.8-72.4]*† | *<0.001* | *0.011* | *<0.001* |
| **Respiratory System Elastance (*cmH_2_O∙kg mL^-1^*)**  0 h  0.5 h | 0.87 [0.82-0.99]  0.85 [0.87-0.98] | 0.93 [0.95-1.0]  0.89 [0.77-1.69]*† | *0.001* | *0.017* | *0.004* |
| **Lung Elastance (*cmH_2_O∙L^-1^*)**  0 h  0.5 h | 18.1 [15.2-21.4]  17.9 [14.2-22.8] | 19.2 [15.8-22.5]*  18.9 [16.1-41.8]*† | *0.005* | *0.003* | *0.010* |
| **Lung Stress (*cmH_2_O*)**  0 h  0.5 h | 7.0 [6.0-8.1]  10.4 [7.6-12.9]† | 6.8 [6.0-7.9]  15.7 [10.5-29.5]*† | *<0.001* | *<0.001* | *<0.001* |
|  | | | | | |
| **Central Venous Pressure *(mmHg)***  0 h  0.5 h | 2 [0-4]  2 [1-3] | 7 [5-9]*  10 [7-12]*† | *<0.001* | *<0.001* | *<0.001* |
| **Mean Pulmonary Artery Pressure *(mmHg)***  0 h  0.5 h | 18 [15-21]  19 [17-26]† | 20 [18-22]  24 [20-32] | *0.001* | *<0.001* | *0.095* |
| **Pulmonary Artery Occlusion Pressure (*mmHg*)**  0 h  0.5 h | 6 [5-7]  6 [5-8] | 9 [7-10]*  12 [9-17]*† | *<0.001* | *<0.001* | *<0.001* |
| **Mean Arterial Pressure (*mmHg*)**  0 h  0.5 h | 77 [73-95]  87 [81-93] | 74 [70-76]  75 [68-83] | *<0.001* | *<0.001* | *0.254* |
| **Cardiac Output *(L·min^-1^·kg^-1^)***  0 h  0.5 h | 0.10 [0.09-0.11]  0.10 [0.09-0.12] | 0.16 [0.15-0.19]*  0.19 [0.16-0.22]*† | *<0.001* | *<0.001* | *<0.001* |
| **Heart Rate (*bpm*)**  0 h  0.5 h | 79 [72-99]  90 [82-112]† | 101 [81-112]*  130 [115-134]*† | *<0.001* | *<0.001* | *<0.001* |
| **Stroke Volume (*mL*)**  0 h  0.5 h | 41 [34-46]  36 [30-41] | 41 [36-46]  37 [29-42] | *0.947* | *<0.001* | *0.305* |

FiO_2_: inspiratory fraction of oxygen; MP_ratio_: mechanical power ratio; PaCO_2_: partial pressure of carbon dioxide; PaO_2_: partial pressure of oxygen.

Variables are expressed as median [IQR]. Changes of physiological variables from 0 (baseline) to 0.5 hour of experimental mechanical power ratio application in high and low mechanical power ratio groups. Two-ways ANOVA analysis: *P_GROUP_* refers to difference between groups; *P_TIME_* refers to change with time; *P_INTER_* refers to different patterns of variables change over time in the two groups. Post-hoc by pairwise Student’s T test: *= *p <0.05* low vs high mechanical power ratio groups; †= *p <0.05* baseline vs 0.5 hour.

***Table E4***. *Physiological variables time-course during the experimental phase*.

| **Groups**  *Pigs Number* | Low MPratio  (≤ 4.5)  **n = 41** | High MPratio  (> 4.5)  **n = 41** | ***p_GROUP_*** | ***p_TIME_*** | ***p_INTER_*** |
| --- | --- | --- | --- | --- | --- |
|  | | | | | |
| **Mechanical Power (*J·min^-1^·kg^-1^*)**  0.5 h  48 h | 0.29 [0.25-0.33]  0.29 [0.23-0.33] | 0.82 [0.56-1.10]*  0.86 [0.62-1.16]* | *<0.001* | *0.051* | *0.002* |
| **Minute Ventilation (*L*∙*min^-1^*∙*kg^-1^*)**  0.5 h  48 h | 0.17 [0.16-0.20]  0.18 [0.15-0.20] | 0.37 [0.24-0.49]  0.35 [0.22-0.49] | *<0.001* | *0.302* | *0.076* |
| **Tidal Volume (*mL*∙*kg^-1^*)**  0.5 h  48 h | 18.2 [10.1-19.6]  17.7 [10.1-19.6] | 14.2 [12.5-30.7]  14.2 [12.3-30.1] | *0.005* | *0.289* | *0.304* |
| **Plateau Pressure (*cmH_2_O*)**  0.5 h  48 h | 18.2 [13.4-20.8]  18.2 [12.5-20.9] | 27.7 [18.3-44.8]*  32.8 [24.0-43.2]* | *<0.001* | *0.068* | *<0.001* |
| **Mean Airway Pressure (*cmH_2_O*)**  0.5 h  48 h | 9 [8-11]  9 [8-10] | 16 [12-30]*  17 [13-28]* | *<0.001* | *0.051* | *0.003* |
| **Driving Pressure (*cmH_2_O*)**  0.5 h  48 h | 13.0 [9.49-15.5]  12.1 [9.37-15.2] | 23.0 [11.9-27.4]*  20.7 [16.2-26.3]* | *<0.001* | *0.300* | *<0.001* |
|  | | | | | |
| **PaO_2_/FiO_2_ (*mmHg*)**  0.5 h  48 h | 512 [412-540]  505 [459-526] | 595 [548-622]*  545 [509-576]*† | *<0.001* | *<0.001* | *<0.001* |
| **PaCO_2_ (*mmHg*)**  0.5 h  48 h | 37.5 [33,42]  33 [29.3,42.8]† | 29.3 [19.5,35.3]*  24.7 [15.8, 29.3]*† | *<0.001* | *<0.001* | *<0.001* |
| **Physiological Dead Space (%)**  0.5 h  48 h | 48 [45-52]  43 [39-47]† | 43 [36-49]*  48 [32-55] | *0.571* | *<0.001* | *0.015* |
| **Venous Admixture (%)**  0.5 h  48 h | 16.8 [14.6-20.7]  17.6 [15.4-20.2] | 8.8 [2.5-14.8]*  6.9 [4.6-11.4]* | *<0.001* | *0.224* | *<0.001* |
|  | | | | | |
| **Respiratory System Elastance (*cmH_2_O·L^-1^*)**  0.5 h  48 h | 29.5 [24.7-35.0]  28.6 [25.2-33.7] | 36.1 [31.7-71.9]*  46.6 [34.4-66.4]* | *<0.001* | *0.039* | *<0.001* |
| **Respiratory System Elastance (*cmH_2_O∙kg mL^-1^*)**  0.5 h  48 h | 0.87 [0.77-0.99]  0.84 [0.77-1.00] | 0.90 [0.77-1.74]  1.29 [0.99-1.58]*† | *<0.001* | *0.035* | *<0.001* |
| **Lung Elastance (*cmH_2_O·L^-1^*)**  0.5 h  48 h | 17.9 [14.2-22.8]  16.7 [12.8-19.5] | 18.7 [16.1-27.9]  27.7 [22.2-38.1]*† | *<0.001* | *0.088* | *<0.001* |
| **Lung Stress (*cmH_2_O*)**  0.5 h  48 h | 10.4 [7.67-13.0]  9.8 [6.75-13.0 | 16.4 [10.7-31.7]*  21.4 [16.7-27.5]* | *<0.001* | *0.401* | *0.002* |
|  | | | | | |
| **Central Venous Pressure**  ***(mmHg)***  0.5 h  48 h | 2 [1-3]  2 [1-3] | 8 [5-11]*  10 [7-14]* | *<0.001* | *<0.001* | *0.006* |
| **Mean Pulmonary Arterial Pressure (*mmHg*)**  0.5 h  48 h | 18 [17-25]  14 [13-15]† | 24 [20-32]*  31 [21-35]* | *<0.001* | *<0.001* | *<0.001* |
| **Pulmonary Arterial Occlusion Pressure (*mmHg*)**  0.5 h  48 h | 6 [5-8]  6 [4-7] | 12 [9-17]  13 [9-19] | *<0.001* | *<0.001* | *0.088* |
| **Mean Arterial Pressure**  **(*mmHg*)**  0.5 h  48 h | 87 [81-92]  66 [59-71]† | 75 [68-82]*  64 [57-71]† | *<0.001* | *<0.001* | *<0.001* |
| **Cardiac Output**  ***(L∙min^-1^∙kg^-1^)***  0.5 h  48 h | 0.10 [0.09-0.12]  0.08 [0.07-0.09]† | 0.19 [0.16-0.22]*  0.14 [0.11-0.20]*† | *<0.001* | *<0.001* | *<0.001* |
| **Heart Rate (*bpm*)**  0.5 h  48 h | 89 [79-107]  70 [61-81]† | 130 [115-154]*  98 [84-120]*† | *<0.001* | *<0.001* | *<0.001* |
| **Stroke Volume (*mL*)**  0.5 h  48 h | 36 [30-42]  35 [28-41] | 37 [29-42]  34 [29-45] | *0.798* | *<0.001* | *0.077* |

FiO_2_: inspiratory fraction of oxygen; MP_ratio_: mechanical power ratio; PaCO_2_: partial pressure of carbon dioxide; PaO_2_: partial pressure of oxygen.

Values are expressed as median [IQR]. Time-course of physiological variables from 0.5 to 48 hours of experimental mechanical power ratio application in high and low mechanical power ratio groups. Two-ways ANOVA analysis: *P_GROUP_* refers to difference between groups; *P_TIME_* refers to change with time; *P_INTER_* refers to different patterns of variables change over time in the two groups. Post-hoc by pairwise Student’s T test: *= *p <0.05* low vs high mechanical power ratio groups; †= *p <0.05* 0.5 vs 48 hour.
